# Supplementary material for: Strategies for retention of heterosexual men in HIV care in sub-Saharan Africa: A systematic review
Source: PLoS One. 2021 Feb 4;16(2):e0246471. doi: 10.1371/journal.pone.0246471 (PMC7861356; doi:10.1371/journal.pone.0246471)
Supplement: S1 Table — (DOCX) [file pone.0246471.s002.docx]

**Supporting information**

**S1 Table. Search term strategy used in Ovid® for three databases (Medline, Embase and Global Health)**

| **Number** | **Search Term** |
| --- | --- |
| 1 | (HIV or HIV-1 or human immunodeficiency virus or human immunodeficiency virus type 1 or human immune deficiency virus or HIV infect* or human immune deficiency virus or human immune deficiency virus or human immun* or deficiency virus or acquired immunodeficiency syndrome* or acquired immune deficiency syndrome or acquired immune deficiency syndrome or acquired immune deficiency syndrome or acquired immun* or deficiency syndrome).af |
| 2 | (HAART or ART or cART or antiretroviral or anti-retroviral or anti-viral or antiviral or Antiretroviral Therapy or Highly Active).af |
| 3 | (Retent* OR adher* or attrition OR loss to follow up OR stay* OR continu* OR confin* OR hold* OR in care OR engag* OR retention OR retain*OR "lost to follow-up" OR "loss to follow-up" OR "loss*" AND "follow up" OR LTFU OR attrition OR "loss to care" OR "lost to care" OR "loss to program* OR "lost to program* OR default* OR engage* OR disengage*).af |
| 4 | (Men or male or man or males OR "adult men" OR "Adult male").af |
| 5 | (sub-Saharan Africa OR Angola OR Benin OR Botswana OR Burkina Faso OR Burundi OR Cameroon OR Cape Verde OR Central African Republic OR Chad OR Comoros OR Congo OR Cote d'Ivoire OR Djibouti OR Equatorial Guinea OR Eritrea OR Ethiopia OR Gabon OR The Gambia OR Ghana OR Guinea OR Guinea-Bissau OR Kenya OR Lesotho OR Liberia OR Madagascar OR Malawi OR Mali OR Mauritania OR Mauritius OR Mozambique OR Namibia OR Niger OR Nigeria OR Reunion OR Rwanda OR "Sao Tome and Principe" OR Senegal OR Seychelles OR Sierra Leone OR Somalia OR South Africa OR Sudan OR Swaziland OR Tanzania OR Togo OR Uganda OR Western Sahara OR Zambia OR Zimbabwe).af |
| 6 | (Randomized controlled trial OR controlled clinical trial OR randomized controlled trials OR random allocation OR random* OR cohort*OR Cohort studies OR Prospective OR Retrospective OR observational).af |
| 7 | 1 and 2 and 3 and 4 and 5 and 6 |
| Limits | Year: 01 January 2005 to 31 April 2019, updated May 2019 to 04 December 2020  Humans |
